# Identifying the significance of nonlinear normal modes

**DOI:** 10.1098/rspa.2016.0789

**Published:** 2017-03

**Authors:** T. L. Hill, A. Cammarano, S. A. Neild, D. A. W. Barton

**Affiliations:** 1 Department of Mechanical Engineering, University of Bristol, Bristol, BS8 1TR, UK; 2 Department of Engineering Mathematics, University of Bristol, Bristol, BS8 1TR, UK; 3 School of Engineering, University of Glasgow, Glasgow, G12 8QQ, UK

**Keywords:** structural dynamics, nonlinear normal modes, backbone curves, energy transfer, resonance, second-order normal form technique

## Abstract

Nonlinear normal modes (NNMs) are widely used as a tool for understanding the forced responses of nonlinear systems. However, the contemporary definition of an NNM also encompasses a large number of dynamic behaviours which are not observed when a system is forced and damped. As such, only a few NNMs are required to understand the forced dynamics. This paper firstly demonstrates the complexity that may arise from the NNMs of a simple nonlinear system—highlighting the need for a method for identifying the significance of NNMs. An analytical investigation is used, alongside energy arguments, to develop an understanding of the mechanisms that relate the NNMs to the forced responses. This provides insight into which NNMs are pertinent to understanding the forced dynamics, and which may be disregarded. The NNMs are compared with simulated forced responses to verify these findings.

## 1. Introduction

Nonlinear normal modes represent an analogue of the established theory of linear normal modes [1] for nonlinear systems. While nonlinear normal modes (NNMs) lack many of the useful mathematical properties of linear normal modes, such as superposition and invariance, they do provide a valuable tool for understanding nonlinear systems [2]. The concept of an NNM was first introduced by Rosenberg in the 1960s [3,4], where it was defined as any *vibration-in-unison* of a conservative nonlinear system—i.e. where the coordinates of the system pass through the equilibrium and reach their extrema simultaneously. Numerous theoretical studies followed this original work [5–8], and NNMs were established as a valuable tool for investigating the behaviour of a variety of nonlinear systems [9,10]. Importantly, it was shown that NNMs (describing the dynamics of the underlying conservative system) could be used to gain an understanding of the resonant forced responses of the system [11].

It has also been demonstrated that some conservative systems may exhibit important dynamic behaviours are not encompassed by the vibration-in-unison definition of an NNM; for example *out-of-unison resonance* [12] where no coordinates reach zero or extrema simultaneously. To allow for a wider variety of dynamic behaviours, the definition of an NNM has since been relaxed to include any *non-necessarily synchronous periodic motion* [2]—i.e. any periodic motion of a conservative nonlinear system is considered to be an NNM. Concurrent work has also extended the NNM concept to damped systems; notably, the work of Shaw & Pierre [13,14], and, more recently, the work of Haller & Ponsioen [15]. This paper focuses on the more common NNM definition, where damping is excluded—i.e. a periodic motion of the underlying conservative system.

The generalization of the conservative NNM definition, along with the development of accessible methods for their computation [16], has led to the use of NNMs in a diverse range of applications in structural dynamics. For example, NNMs have been used to understand the dynamic behaviour of full-scale aircraft and satellites [17,18], as a tool in substructuring [19] and the construction of reduced-order models [20], and for damage detection in engineering structures [21]. Along with such large-scale mechanical structures, NNMs have also seen successful application to MEMS devices [22], nanostructures [23] and to acoustic–structure interactions [24].

A key feature of NNMs is their relationship to the resonant forced responses of the system, providing an understanding of the forced behaviour [25] and enabling the prediction of important features in forced responses, such as isolas [26–28]. However, in [29]—which explores the dynamics of a taut, horizontal cable—it was observed that, for the forcing and damping cases that are considered, not all sets of NNMs relate to forced responses. This suggests that not all sets of NNMs (also referred to as *NNM branches* or *backbone curves*) are of equal significance when considering the forced responses. Determining the nature of this significance is the primary focus of this paper.

To motivate discussion throughout this paper, we consider a pinned-pinned beam, with a torsional spring at one end. In line with much of the literature, the system is expressed in terms of the linear modes (i.e. the modes of the underlying linear system) resulting in differential equations with nonlinear coupling terms [30]. Here, a nonlinear model consisting of the first two underlying linear modes is used.^1^ Numerical continuation reveals that this simple model exhibits a vast array of NNM branches. The abundance of branches depicts a complex picture of the dynamic behaviours, and determining which NNMs are pertinent to understanding the forced responses becomes challenging. This highlights the necessity for a technique that allows the relative significance of NNMs to be quantified, allowing the complex range of behaviours to be reduced to those that directly relate to the forced dynamics.

In §3, an analytical approach is used to describe the NNM branches and to gain an understanding of the physical mechanisms that lead to the dynamic behaviours represented by the NNMs. It is shown that some NNM branches exhibit *phase-locking*, where a specific phase relationship exists between the fundamental components of the linear modes, while other NNM branches do not exhibit phase-locking, referred to as *phase-unlocked* branches. Section 4 then considers the phase difference between the two linear modes of the model. Firstly, a numerical approach is used to investigate the case where the system is forced such that the response lies on an NNM branch. A perturbation is then applied to the phase of the external forcing, which results in a deviation of the response from the NNM branch. It is found that the magnitude of this deviation varies significantly between the different NNM branches, and hence is used as a means of quantifying the *attraction* of the forced response to the NNM. The NNM branches that exhibit little attraction may be considered less significant than those with a greater attraction.

While this numerical approach gives insight into the relative significance of the NNMs, it does not reveal the mechanisms that cause (or prevent) the attraction between the forced responses and the NNMs. This is explored in §4b where the analytical results, derived in §3, are used to show that energy may only be transferred between the fundamental components of the linear modes in phase-locked responses. This ability to transfer energy between the modes is required for the majority of forced responses, as discussed in [25]. For the phase-unlocked NNMs, some energy may still be transferred between the linear modes via changes in the phase difference between the harmonics. However, as the harmonics are typically small compared with the fundamental components, this energy transfer mechanism is far weaker than that of the phase-locked NNMs. As such, phase-unlocked NNMs are unlikely to achieve the necessary energy transfer in the presence of forcing.

In §5, the response of the system, subjected to a sinusoidal external forcing, is demonstrated. Initially, a forcing and damping level that is typical of many engineering systems is considered, and it is seen that the responses follow the phase-locked NNM branches. Next, the damping level is significantly reduced. It is argued that, due to the very low damping, a weak energy transfer mechanism between the modes may be sufficient for an NNM to be attractive, and the phase-unlocked NNMs may be able to provide this via the harmonics. This is confirmed using numerical continuation results, which show that a section of the forced response follows a phase-unlocked branch. While this demonstrates that phase-unlocked branches may represent dynamic behaviours that underpin the forced responses, it is concluded that they are only significant for structures with extremely low levels of damping.

## 2. Periodic responses of a nonlinear beam

The contemporary definition of a NNM encompasses any periodic motion of a system [2]. In this section, we consider a simple nonlinear system—represented in the form of two cross-coupled linear modal equations—and demonstrate how it may exhibit an extremely large number of responses that satisfy this definition. Such an abundance of NNM responses presents a challenge when using the NNMs to gain insight into the behaviour of the forced responses. This motivates the discussion for subsequent sections, where the relationship between the NNMs and the forced responses of this structure are explored, and the relative significance of the NNMs, in relation to the forced responses, are determined.

**Figure 1.**
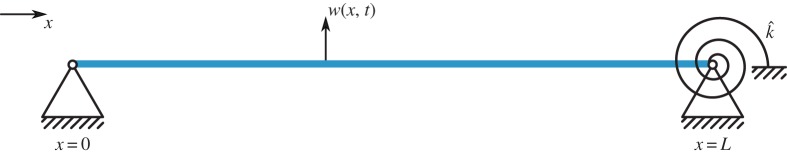
Schematic of a pinned-pinned beam with a rotational constraint at one end. (Online version in colour.)

The pinned-pinned beam represented in figure 1 is used as a motivating example throughout this paper. The distance along this beam is denoted *x* and at *x*=*L*, where *L* is the length of the beam, there is a rotational spring breaking the symmetry of the system. The axial tension in the beam is assumed to be zero at equilibrium; however, owing to the pinned-pinned boundary conditions, a significant dynamic axial tension is present when the beam is deflected vertically, denoted *w*(*x*,*t*). From [31], the motion of this beam may be described by

2.1 $$\rho \hat{A}\frac{\partial^{2}w(x,t)}{\partial t^{2}}+EI\frac{\partial^{4}w(x,t)}{\partial x^{4}}-\;\left[\frac{E\hat{A}}{2L}\int_{0}^{L}{\left(\frac{\partial w(x,t)}{\partial x}\right)}^{2}\;dx\right]\frac{\partial^{2}w(x,t)}{\partial x^{2}}+\delta (x-L)\hat{k}\psi (L,t)=0,$$

where *δ* denotes the Dirac delta function, *ψ*(*x*,*t*) is the rotation of the beam and the constants *ρ*, $\hat{A}$, *E* and *I* represent the density, cross-sectional area, Young’s modulus and second moment of area of the beam, respectively. The term in the square brackets represents the dynamic tension in the beam and this results in a nonlinear component in the equation of motion, equation (2.1).

The dynamics of the beam is modelled using the first two linear modes and, for simplicity, all higher modes are neglected. As such, the vertical deflection may be written as

2.2 $$w(x,t)=\theta_{1}(x)q_{1}(t)+\theta_{2}(x)q_{2}(t),$$

where *θ*_*i*_ and *q*_*i*_ represent the modeshape and displacement of the *i*th linear mode, respectively. The modeshapes may be computed using the expression provided in appendix A—see equation (.1). As detailed in [32], Galerkin’s method may be used to write the equation of motion of the displacements of the first two modes as

2.3 $$\ddot{\text{q}}+\Lambda \text{q}+{\text{N}}_{q}(\text{q})=0.$$

For the system considered here, the elements in equation (2.3) are written

2.4 $$\text{q}=\left(\begin{array}{c} q_{1}\\ q_{2} \end{array}\right),\;\Lambda =\left[\begin{array}{cc} \omega_{n1}^{2} & 0\\ 0 & \omega_{n2}^{2} \end{array}\right],\;{\text{N}}_{q}=\left(\begin{array}{c} \alpha_{1}q_{1}^{3}+3\alpha_{2}q_{1}^{2}q_{2}+\alpha_{3}q_{1}q_{2}^{2}+\alpha_{4}q_{2}^{3}\\ \alpha_{2}q_{1}^{3}+\alpha_{3}q_{1}^{2}q_{2}+3\alpha_{4}q_{1}q_{2}^{2}+\alpha_{5}q_{2}^{3} \end{array}\right),$$

where *ω*_*ni*_ is the natural frequency of the *i*th linear mode and *α*_*k*_ are nonlinear coefficients. These may be found using equations (A 3) and (A 4).

The beam considered throughout this paper has a length, depth and height of *L*=500 mm, *d*=30 mm and *h*=1 mm, respectively. The beam also has a density and Young’s modulus of *ρ*=7800 kg m^−3^ and *E*=2×10^11^ N m^−2^, respectively, and the stiffness of the torsional spring is $\hat{k}=10\;N\;m\;{\mathrm{rad}}^{-1}$. This gives the natural frequencies and nonlinear parameters shown in table 1.

**Table 1.** The linear natural frequencies, *ω*_*ni*_, and nonlinear parameters, *α*_*k*_, of the beam.

*ω*_*n*1_	*ω*_*n*2_	*α*_1_	*α*_2_	*α*_3_	*α*_4_	*α*_5_
	(rad s^−1^)			(×10^10^)		
125.91	418.41	8.81	−1.31	34.70	−5.12	133.63

### (a) A branch of nonlinear normal modes

**Figure 2.**
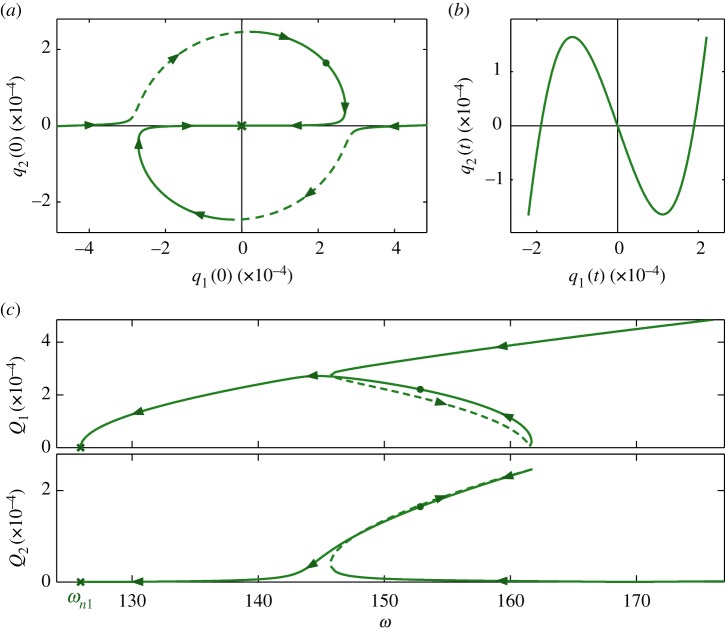
A set of NNM branches of the example system, represented in different projections: panel (*a*) shows the initial displacements of the linear modes, *q*_1_(0) against *q*_2_(0), when the initial modal velocities are zero; panel (*c*) shows the base frequency, *ω*, against the maximum amplitude of displacement of the first mode, *Q*_1_, and second mode, *Q*_2_; and panel (*b*) shows a single NNM, parametrized in time, in terms of the displacements of the two modes, *q*_1_ against *q*_2_. The unstable section of this branch is represented by a dashed line. The NNM shown in (*b*) is represented by a dot on each of the curves in (*a*) and (*c*). Note that, while this resembles the forced response of a nonlinear system, this figure is showing the locus of unforced, undamped periodic responses (i.e. NNMs). Additionally, the physical parameters of the system are constant for all NNMs represented by these branches; however, the *energy* of the system varies between NNMs. (Online version in colour.)

Figure 2 shows a set of NNMs—i.e. periodic solutions to equation (2.3)—that have been computed using the numerical continuation software AUTO-07p [33]. Figures 2*a*,*c* represent this NNM branch in three different projections. Figure 2*a* shows the initial displacement of the first mode, *q*_1_(0), against the initial displacement of the second mode *q*_2_(0); the top panel of figure 2*c* shows the maximum displacement amplitude of the first mode, *Q*_1_, in terms of the *base frequency* *ω*, where *ω*=2*π*/*T* and where *T* is the period; and the bottom panel of figure 2*c* shows the base frequency, *ω*, against the maximum displacement amplitude of the second mode, *Q*_2_. Figure 2*b* shows one particular NNM, parametrized in time, in the projection of the modal displacements, *q*_1_ against *q*_2_. This response corresponds to the points marked with a dot on each of the curves in figures 2*a*,*c*. In all conservative responses that are considered in this paper, the initial velocities are zero, such that they represent the responses of the system when released from rest.

In figures 2*a*,*c* crosses are used to show where *q*_1_(0)=*q*_2_(0)=0. It can be seen that this corresponds to the point at which the base frequency is equal to the first linear natural frequency, i.e. *ω*=*ω*_*n*1_. This is due to the behaviour of this system tending to that of the underlying linear system at low-amplitude and, as such, this may be seen as a nonlinear extension of the first linear mode. Figures 2*a*,*c* also show a series of arrowheads, intended to aid comparisons between these panels. It can also be seen that figure 2*a* is rotationally symmetric about [0,0]—a result of the symmetry in the restoring forces for this system, as only linear and cubic terms are present, i.e. there are no even stiffness components. Owing to the symmetry, and as amplitude is positive, the two sets of arrows shown in figure 2*a* are superposed in figure 2*c*.

For the NNM represented in figure 2*b*, the response frequency of the second linear mode is three times that of the first. This behaviour is observed for the majority of NNMs represented by this branch shown in figures 2*a*,*c*, and hence this is denoted the 1 : 3 NNM branch. Strictly, these responses also contain harmonic components; however, the frequency of interest is that of the component with the highest amplitude, known as the *fundamental component*. The fundamental frequency of the *i*th mode is denoted as *ω*_*ri*_ and is related to the base frequency by *ω*_*ri*_=*r*_*i*_*ω*, where *r*_*i*_ is an integer.^2^ For the NNM shown in figure 2*b*, the fundamental response frequency for the first and second modes respond at one and three times the base frequency, i.e. *r*_1_=1 and *r*_2_=3.

### (b) Additional nonlinear normal mode branches

**Figure 3.**
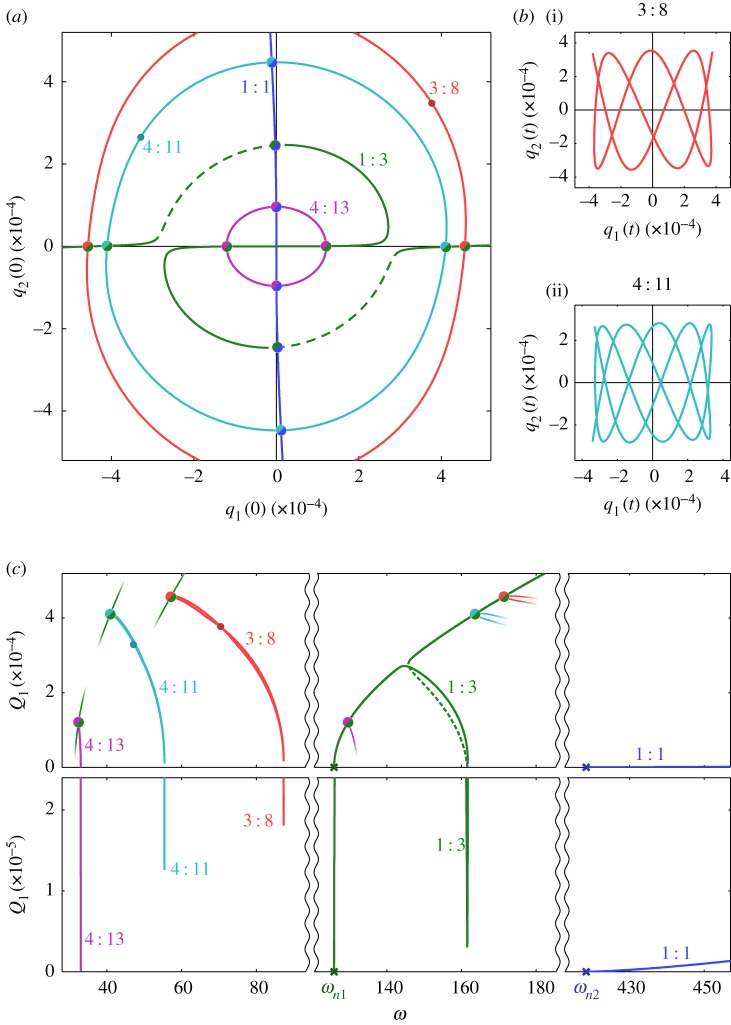
Five NNM branches for the example system, represented in a number of different projections. In panel (*a*) these branches are shown in the projection of the initial displacements of the linear modes, *q*_1_(0) against *q*_2_(0). The marginally stable and unstable sections are represented by solid and dashed lines, respectively. Large dots are used to mark the intersections between the NNM branches, and the colours of these dots correspond to the colours of the two branches. Panel (*c*) shows these branches in the projection of the base frequency, *ω*, against the maximum displacement amplitude of the first linear mode, *Q*_1_. Note that panel (*c*) shows this projection in two different amplitude ranges, with the bottom section showing the low-amplitude responses in detail. Large dots are used to mark the branch intersections seen in panel (*a*) and small segments show the intersecting branches. The base frequencies of these branches are scaled with the change in period between the branches. Panels (*b*(i)) and (*b*(ii)) both show individual NNMs, parametrized in time, in the projection of the displacements of the two modes, *q*_1_ against *q*_2_. Panels (*b*(i)) and (*b*(ii)) show NNMs on the 3 : 8 and 4 : 11 branches, respectively, and the locations of these NNMs are represented by small dots in panels (*a*) and (*c*). (Online version in colour.)

Figure 3 shows additional NNM branches for this system. As with figure 2*a*, figure 3*a* shows these branches in the projection of the initial displacements of the modes, *q*_1_(0) against *q*_2_(0), where the initial velocities of both modes are zero. This plot shows five different branches, computed using numerical continuation and labelled 1 : 1, 1 : 3, 3 : 8, 4 : 11 and 4 : 13 (with *n*:*m* denoting the ratio between the fundamental response frequencies). Note that the 1 : 3 branch was previously shown in figure 2*a* and is repeated here.

It can be seen that many of these NNM branches intersect, at points represented by large dots, and hence must share responses at these points—for example the 1 : 3 and 3 : 8 curves meet near *q*_2_(0)=0. As an NNM cannot have a fundamental response frequency ratio of both 1 : 3 and 3 : 8 simultaneously, this demonstrates that these ratios cannot be true for all points on the curves. However, the regions where these ratios are incorrect are small and hence the branches are labelled according to the frequency ratio that is observed for the vast majority of responses represented by the curve. The intersections between the curves are also associated with a change in period. For example, it is found that, as the 3 : 8 branch approaches the intersection with 1 : 3, the component in the second mode responding at 9*ω* grows, while the component at 8*ω* diminishes (where *ω* is the base frequency). At the point of intersection, the component at 8*ω* has zero amplitude and the NNM may be described as having a response frequency ratio of 3 : 9. Furthermore, all components at *kω*, where *k* is not divisible by three, are zero, and this NNM is identical to that of the NNM on the 1 : 3 branch at this point, but repeats three times. As such, the transition from the 1 : 3 to the 3 : 8 curve is associated with a tripling in the period.

**Figure 4.**
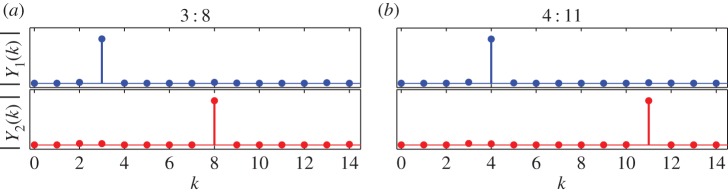
The amplitudes of the first 15 Fourier components of two different NNMs—one from the 3 : 8 branch and one from the 4 : 11 branch. These NNMs were shown previously in figures 3*b*(i) and 3*b*(ii), respectively, parametrized in time. Panels (*a*) and (*b*) are each separated into two sections: the top section showing the amplitude of the *kth* Fourier component (i.e. the component responding at *kω*) for the first modal displacement, *q*_1_(*t*), denoted $|Y_{1}(k)|$; and the bottom section showing the components of the second modal displacement, *q*_2_(*t*), denoted $|Y_{2}(k)|$. (Online version in colour.)

The small dots on the 3 : 8 and 4 : 11 branches in figure 3*a* mark two NNMs that are shown, parametrized in time, in figures 3*b*(i) and 3*b*(ii), respectively. These are both represented in the projection of the first modal displacement, *q*_1_, against the second modal displacement, *q*_2_, and highlight the complexity of these responses. The amplitudes of the Fourier components of these NNMs are shown in figure 4.

Figure 3*c* shows the NNM branches in terms of the base frequency and displacement amplitudes, as used previously in figure 2*c*. It can be seen that the branches, which intersect in figure 3*a*, no longer intersect in this projection, due to the change in period associated with these crossing points. Instead, small segments of the branches are shown, where the base frequency has been adjusted to reflect the change in period at the intersection. For example, the segments of the 1 : 3 (green) branch intersecting with the 4 : 11 (cyan) and 4 : 13 (purple) branches are shown at *ω*/4, and the segment of the 3 : 8 (red) branch intersecting with the 1 : 3 (green) branch is shown at 3*ω*. Similar intersections in the frequency projection may be observed with the 1 : 1 branch; however, for simplicity, these are not shown here.

**Figure 5.**
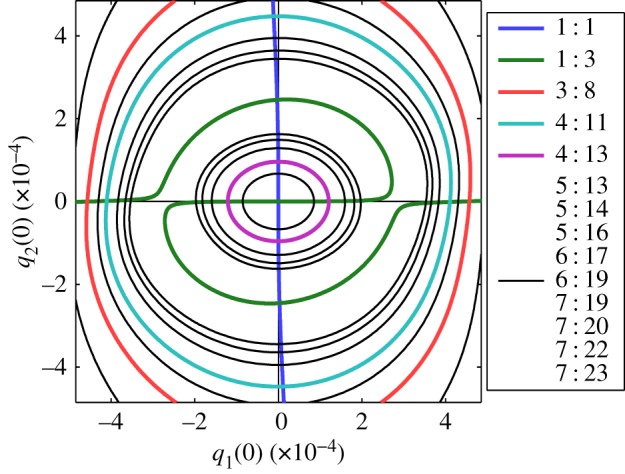
A further set of NNM branches, represented in the projection of the initial conditions of the modal displacements, *q*_1_(0) against *q*_2_(0). The branches that were previously shown in figure 3 are also shown here, and the corresponding colours are used. Additional branches, with higher response frequency ratios, are represented by thin black lines. (Online version in colour.)

Figure 3 demonstrates the abundance of NNM branches in this nonlinear system; however, this does not show all branches for this system. As demonstrated in figure 5, many more branches may be observed if higher frequency ratios are considered. Furthermore, additional branches exist in regions beyond that shown here, and more branches, with higher response frequency ratios (associated with longer periods), exist within this region. As such, this figure is not intended as a complete picture of these responses, but simply as a demonstration of their abundance and the complexity that arises from NNMs with high fundamental frequency ratios.

NNMs are often used as a means of simplifying the responses of a system in order to gain insight into the underlying dynamic behaviour [2] and to predict the forced responses [25]. However, the abundance of responses shown in figure 5 appears to call into question the efficacy of NNMs. Because such a large and complex range of NNMs exist, it is important to identify which NNMs correspond to dynamics that are observed in the presence of external forcing and damping. Without an understanding of this relationship, the complex picture given by NNMs cannot be simplified, and their use as a tool for interpreting forced responses is limited. As such, the remainder of this paper explores the relationship between the NNMs and the forced responses, the mechanisms that govern this relationship, and how the relationship may be quantified.

## 3. An analytical investigation of the two-mode beam model

In this section, an analytical method is used to describe these NNM responses. This gives insight into the physical mechanisms that underpin the dynamics of each of the NNM branches in the two-mode beam model. The second-order normal form technique is adopted here [34], as this method allows expressions for the harmonics to be computed—as demonstrated in §4c.

### (a) The second-order normal form technique

The conservative system considered here is expressed in terms of its linear modal coordinates with no linear coupling between the modes. As such, the first two steps of the second-order normal form technique (the linear and forcing transforms) are omitted. For a complete description of the technique see [34], and for the application of the technique to similar systems to that considered here, see [29,35].

For the system considered here, the second-order normal form technique consists only of the nonlinear near-identity transform. This transform takes the form **q**=**u**+**h**(**u**), where **u** and **h** represent the fundamental and harmonic components of the modal displacements, **q**, respectively. Note that it is assumed that the harmonics are small in comparison with the fundamental components, and may be expressed as a function of the fundamental components. The aim of this transform is to obtain an expression for the resonant equation of motion, written

3.1 $$\ddot{\text{u}}+\Lambda \text{u}+{\text{N}}_{u}(\text{u})=0,$$

where **N**_*u*_ is a vector of resonant nonlinear terms, such that all terms in the *i*th element of **N**_*u*_ respond at the *i*th fundamental response frequency, *ω*_*ri*_. As equation (3.1) contains only resonant nonlinear terms, it may be solved to find **u** using

3.2 $$u_{i}=U_{i}\cos(\omega_{ri}t-\phi_{i})=\frac{U_{i}}{2}e^{+j(\omega_{ri}t-\phi_{i})}+\frac{U_{i}}{2}e^{-j(\omega_{ri}t-\phi_{i})}=u_{pi}+u_{mi},$$

where *U*_*i*_, *ω*_*ri*_ and *ϕ*_*i*_ represent the amplitude, response frequency and phase of the fundamental component of the *i*th mode, respectively. Note that the subscripts of *u*_*pi*_ and *u*_*mi*_ denote the positive and negative (*plus* and *minus*) signs of the exponents, respectively. As the harmonics **h** are expressed in terms of **u**, the harmonics may be found by substituting the solutions for **u**.

As shown in [34], the first step in finding the transform **q**=**u**+**h** is to substitute the approximation **q**=**u** into the vector of nonlinear modal terms, **N**_*q*_(**q**). From equation (2.4), this gives

3.3 $$\begin{array}{rl} {\text{N}}_{q}(\text{u}) & =(\begin{array}{c} \alpha_{1}{\left(u_{p1}+u_{m1}\right)}^{3}+3\alpha_{2}{\left(u_{p1}+u_{m1}\right)}^{2}(u_{p2}+u_{m2})\\ \alpha_{2}{\left(u_{p1}+u_{m1}\right)}^{3}+\alpha_{3}{\left(u_{p1}+u_{m1}\right)}^{2}(u_{p2}+u_{m2}) \end{array}\\ & \;\;\begin{array}{c} +\;\alpha_{3}(u_{p1}+u_{m1}){\left(u_{p2}+u_{m2}\right)}^{2}+\alpha_{4}{\left(u_{p2}+u_{m2}\right)}^{3}\\ +\;3\alpha_{4}(u_{p1}+u_{m1}){\left(u_{p2}+u_{m2}\right)}^{2}+\alpha_{5}{\left(u_{p2}+u_{m2}\right)}^{3} \end{array}), \end{array}$$

where, from equation (3.2), the substitution *u*_*i*_=*u*_*pi*_+*u*_*mi*_ has been made. The brackets in equation (3.3) may then be expanded to give 20 unique monomials in each vector element.

Next, **N**_*q*_ is expressed as the product **N**_*q*_=[*N*_*q*_]u* where [*N*_*q*_] is a {2×20} matrix of coefficients and u* is a {20×1} vector of variables, whose ℓ*th* element may be written as

3.4 $$u_{\ell }^{*}=u_{p1}^{s_{p\ell 1}}u_{m1}^{s_{m\ell 1}}u_{p2}^{s_{p\ell 2}}u_{m2}^{s_{m\ell 2}},$$

where *s*_*p*ℓ*k*_ and *s*_*m*ℓ*k*_ are the exponents of *u*_*pk*_ and *u*_*mk*_, respectively. As all time-varying terms are contained within the vector u*, it may be used to determine which nonlinear terms are resonant, and thus which appear in the resonant equation of motion, equation (3.1). This is achieved using the {20×2} matrix ***β***, which is computed from

3.5 $$\beta_{i,\ell }={\left(\sum_{k=1}^{2}[s_{p\ell k}-s_{m\ell k}]\omega_{rk}\right)}^{2}-\omega_{ri}^{2}=\left\{{\left(\sum_{k=1}^{2}[s_{p\ell k}-s_{m\ell k}]r_{k}\right)}^{2}-r_{i}^{2}\right\}\omega^{2},$$

where *β*_*i*,ℓ_ represents element {*i*,ℓ} of ***β*** and where the base frequency, *ω*=2*π*/*T*, has been used to express the fundamental response frequencies as *ω*_*ri*_=*r*_*i*_*ω*. Now, using equations (3.3)–(3.5), [*N*_*q*_], u* and ***β*** may be computed as

3.6 $${\left[N_{q}\right]}^{⊺}=\left[\begin{array}{cc} \alpha_{1} & \alpha_{2}\\ 3\alpha_{1} & 3\alpha_{2}\\ 3\alpha_{1} & 3\alpha_{2}\\ \alpha_{1} & \alpha_{2}\\ 3\alpha_{2} & \alpha_{3}\\ 6\alpha_{2} & 2\alpha_{3}\\ 3\alpha_{2} & \alpha_{3}\\ 3\alpha_{2} & \alpha_{3}\\ 6\alpha_{2} & 2\alpha_{3}\\ 3\alpha_{2} & \alpha_{3}\\ \alpha_{3} & 3\alpha_{4}\\ 2\alpha_{3} & 6\alpha_{4}\\ \alpha_{3} & 3\alpha_{4}\\ \alpha_{3} & 3\alpha_{4}\\ 2\alpha_{3} & 6\alpha_{4}\\ \alpha_{3} & 3\alpha_{4}\\ \alpha_{4} & \alpha_{5}\\ 3\alpha_{4} & 3\alpha_{5}\\ 3\alpha_{4} & 3\alpha_{5}\\ \alpha_{4} & \alpha_{5} \end{array}\right],\;{\text{u}}^{*}=\left[\begin{array}{c} u_{p1}^{3}\\ u_{p1}^{2}u_{m1}\\ u_{p1}u_{m1}^{2}\\ u_{m1}^{3}\\ u_{p1}^{2}u_{p2}\\ u_{p1}u_{m1}u_{p2}\\ u_{m1}^{2}u_{p2}\\ u_{p1}^{2}u_{m2}\\ u_{p1}u_{m1}u_{m2}\\ u_{m1}^{2}u_{m2}\\ u_{p1}u_{p2}^{2}\\ u_{p1}u_{p2}u_{m2}\\ u_{p1}u_{m2}^{2}\\ u_{m1}u_{p2}^{2}\\ u_{m1}u_{p2}u_{m2}\\ u_{m1}u_{m2}^{2}\\ u_{p2}^{3}\\ u_{p2}^{2}u_{m2}\\ u_{p2}u_{m2}^{2}\\ u_{m2}^{3} \end{array}\right],\;\frac{\beta^{⊺}}{\omega_{r1}^{2}}=\left[\begin{array}{cc} 8 & 9-r^{2}\\ 0 & 1-r^{2}\\ 0 & 1-r^{2}\\ 8 & 9-r^{2}\\ \left(r+1)(r+3\right) & 4(1+r)\\ r^{2}-1 & 0\\ \left(r-1)(r-3\right) & 4(1-r)\\ \left(r-1)(r-3\right) & 4(1-r)\\ r^{2}-1 & 0\\ \left(r+1)(r+3\right) & 4(1+r)\\ 4r(r+1) & \left(3r+1)(r+1\right)\\ 0 & 1-r^{2}\\ 4r(r-1) & \left(3r-1)(r-1\right)\\ 4r(r-1) & \left(3r-1)(r-1\right)\\ 0 & 1-r^{2}\\ 4r(r+1) & \left(3r+1)(r+1\right)\\ 9r^{2}-1 & 8r^{2}\\ r^{2}-1 & 0\\ r^{2}-1 & 0\\ 9r^{2}-1 & 8r^{2} \end{array}\right],$$

where *r*=*r*_2_/*r*_1_ such that *ω*_*r*2_=*rω*_*r*1_.

In order to find the resonant nonlinear terms, **N**_*u*_, and the harmonic terms, **h**, the {20×2} coefficient matrices [*N*_*u*_] and [*h*] are defined using **N**_*u*_=[*N*_*u*_]u* and **h**=[*h*]u*. As discussed in [34], these matrices are populated with the coefficients from [*N*_*q*_], such that the coefficients of the resonant terms are transferred to the matrix of resonant coefficients, [*N*_*u*_], and the non-resonant coefficients are transferred to the matrix of harmonic coefficients, [*h*]. This may be expressed [*N*_*q*_]=[*N*_*u*_]+***β***°[*h*], where ° represents the Hadamard product. From this, the value of element {*i*,ℓ} in the matrices [*N*_*u*_] and [*h*] may be found using

3.7 $${\left[N_{u}\right]}_{i,\ell }=\left\{ \begin{array}{cc} {\left[N_{q}\right]}_{i,\ell } & \mathrm{if}:\;\beta_{i,\ell }=0,\\ 0 & \mathrm{if}:\;\beta_{i,\ell }\neq 0, \end{array}\right.\;{\left[h\right]}_{i,\ell }=\left\{ \begin{array}{cc} 0 & \mathrm{if}:\;\beta_{i,\ell }=0,\\ \frac{{\left[N_{q}\right]}_{i,\ell }}{\beta_{i,\ell }} & \mathrm{if}:\;\beta_{i,\ell }\neq 0\;. \end{array}\right.$$

As can be seen from equation (3.6), and as discussed in [32,35], there are three types of terms represented by ***β***: *unconditionally resonant* terms, which are resonant regardless of the value of *r*; *conditionally resonant* terms, which are only resonant for specific values of *r*; and *non-resonant* terms, which cannot be resonant. It can therefore be determined, from equation (3.7), that the coefficients of unconditionally resonant terms will always appear in [*N*_*u*_] but never in [*h*], while the non-resonant coefficients will always appear in [*h*] but never in [*N*_*u*_], and the conditionally resonant terms may appear in either but not both. For the system considered here, the conditionally resonant terms are only resonant when $r=\frac{1}{3}$, *r*=1 or *r*=3.^3^ It is found that the harmonics of the $r=\frac{1}{3}$ solutions are larger than the fundamental components, which violates an assumption of the second-order normal form technique. As such, it is determined that the $r=\frac{1}{3}$ solutions are not valid and hence this case is not presented here.

Using the general form of the resonant equation of motion, equation (3.1), along with the assumed solution for *u*_*i*_, equation (3.2), the *i*th resonant equation of motion may be written as

3.8 $$\left[(\omega_{ni}^{2}-r_{i}^{2}\omega^{2})\frac{U_{i}}{2}+N_{ui}^{+}\right]e^{+j(\omega_{ri}t-\phi_{i})}+\left[(\omega_{ni}^{2}-r_{i}^{2}\omega^{2})\frac{U_{i}}{2}+N_{ui}^{-}\right]e^{-j(\omega_{ri}t-\phi_{i})}=0,$$

where $N_{ui}^{+}$ and $N_{ui}^{-}$ are time-independent complex conjugates. As the contents of both sets of square brackets in equation (3.8) are time-independent, they may each be equated to zero, i.e.

3.9 $$(\omega_{ni}^{2}-r_{i}^{2}\omega^{2})\frac{U_{i}}{2}+N_{ui}^{+}=0.$$

To find the nonlinear components, $N_{ui}^{+}$, equations (3.6) and (3.7) are used, along with the definition **N**_*u*_=[*N*_*u*_]u*, to give

3.10a $$\begin{array}{rl} N_{u1}^{+} & =\frac{1}{8}\{3\alpha_{1}U_{1}^{3}+2\alpha_{3}U_{1}U_{2}^{2}+\delta_{3}[3\alpha_{2}U_{1}^{2}U_{2}e^{+j{\hat{\phi }}_{3,1}}]\\ & \;+\delta_{1}[3\alpha_{2}U_{1}^{2}U_{2}(2e^{+j{\hat{\phi }}_{1,1}}+e^{-j{\hat{\phi }}_{1,1}})+\alpha_{3}U_{1}U_{2}^{2}e^{+j2{\hat{\phi }}_{1,1}}+3\alpha_{4}U_{2}^{3}e^{+j{\hat{\phi }}_{1,1}}]\} \end{array}$$

and

3.10b $$\begin{array}{rl} N_{u2}^{+} & =\frac{1}{8}\{2\alpha_{3}U_{1}^{2}U_{2}+3\alpha_{5}U_{2}^{3}+\delta_{3}[\alpha_{2}U_{1}^{3}e^{-j{\hat{\phi }}_{3,1}}]\\ & \;+\delta_{1}[3\alpha_{4}U_{1}U_{2}^{2}(2e^{-j{\hat{\phi }}_{1,1}}+e^{+j{\hat{\phi }}_{1,1}})+\alpha_{3}U_{1}^{2}U_{2}e^{-j2{\hat{\phi }}_{1,1}}+3\alpha_{2}U_{1}^{3}e^{-j{\hat{\phi }}_{1,1}}]\}, \end{array}$$

where the phase difference, ${\hat{\phi }}_{i,j}$, is defined as ${\hat{\phi }}_{i,j}=i\phi_{1}-j\phi_{2}$ and where *δ*_*i*_ is defined by

3.11 $$\delta_{i}=\left\{ \begin{array}{ll} 1 & \mathrm{if}:\;i=r,\\ 0 & \mathrm{if}:\;i\neq r, \end{array}\right.$$

such that *δ*_*i*_ corresponds to the conditionally resonant terms. As the $r=\frac{1}{3}$ case does not lead to valid solutions, the conditionally resonant terms resulting from $r=\frac{1}{3}$ have not been included.

As previously discussed, the terms in equations (3.6) may be categorized as either unconditionally resonant, non-resonant or conditionally resonant. Equation (3.10) also reveals that only the conditionally resonant terms are phase-dependent, i.e. those containing a *δ*_*i*_ component, also contain a ${\hat{\phi }}_{i,j}$ component. This follows from [32], where it is shown that only conditionally resonant terms may lead to a phase relationship in the resonant equations of motion.^4^

### (b) Computing the nonlinear normal mode branches

The resonant equations of motion, equations (3.9) and (3.10), are now used to compute the NNM branches, or *backbone curves*, of the system. As previously discussed, this paper only considers responses with an initial velocity of zero. Therefore, neglecting harmonics, the initial velocity of the *i*th mode may be written ${\dot{u}}_{i}(0)=\omega_{ri}U_{i}\sin(\phi_{i})=0$, from equation (3.2). From this, it may be determined that *ϕ*_*i*_=0,*π* such that the phase difference is ${\hat{\phi }}_{i,j}=i\phi_{1}-j\phi_{2}=0,\pi$. This allows the variable *p* to be introduced, where

3.12 $$p=e^{+j{\hat{\phi }}_{i,j}}=e^{-j{\hat{\phi }}_{i,j}}=\left\{ \begin{array}{ll} +1 & \mathrm{when}:\;i\phi_{1}-j\phi_{2}=0,\\ -1 & \mathrm{when}:\;i\phi_{1}-j\phi_{2}=\pi , \end{array}\right.$$

i.e. *p*=+1 when the two modes are in-phase and *p*=−1 when the two modes are in anti-phase.

For the case, where *r*_1_=*r*_2_=1, such that *r*=1, the resonant equations of motion may be found by combining equations (3.9) and (3.10) to give

3.13a $$4(\omega_{n1}^{2}-\omega^{2})U_{1}+3\alpha_{1}U_{1}^{3}+2\alpha_{3}U_{1}U_{2}^{2}+9p\alpha_{2}U_{1}^{2}U_{2}+\alpha_{3}U_{1}U_{2}^{2}+3p\alpha_{4}U_{2}^{3}=0$$

and

3.13b $$4(\omega_{n2}^{2}-\omega^{2})U_{2}+2\alpha_{3}U_{1}^{2}U_{2}+3\alpha_{5}U_{2}^{3}+9p\alpha_{4}U_{1}U_{2}^{2}+\alpha_{3}U_{1}^{2}U_{2}+3p\alpha_{2}U_{1}^{3}=0,$$

where equation (3.12) has been substituted. Assuming *U*_1_≠0 and *U*_2_≠0, equations (3.13) may be written as

3.14a $$\omega^{2}=\omega_{n1}^{2}+\frac{1}{4}[3\alpha_{1}U_{1}^{2}+3\alpha_{3}U_{2}^{2}+9p\alpha_{2}U_{1}U_{2}+3p\alpha_{4}U_{1}^{-1}U_{2}^{3}]$$

and

3.14b $$\omega^{2}=\omega_{n2}^{2}+\frac{1}{4}[3\alpha_{3}U_{1}^{2}+3\alpha_{5}U_{2}^{2}+9p\alpha_{4}U_{1}U_{2}+3p\alpha_{2}U_{1}^{3}U_{2}^{-1}],$$

which may be combined to give the amplitude relationship

3.15 $$\begin{array}{rl} & [-3p\alpha_{2}U_{2}^{-1}]U_{1}^{4}+[3(\alpha_{1}-\alpha_{3})]U_{1}^{3}+[9p(\alpha_{2}-\alpha_{4})U_{2}]U_{1}^{2}\\ & \;+[4(\omega_{n1}^{2}-\omega_{n2}^{2})+3(\alpha_{3}-\alpha_{5})U_{2}^{2}]U_{1}+[3p\alpha_{4}U_{2}^{3}]=0. \end{array}$$

Equations (3.15) may be solved to find the amplitude *U*_1_ in terms of the amplitude *U*_2_ for both the in- and anti-phase cases (i.e. where *p*=+1 and *p*=−1, respectively). These solutions may then be substituted into equations (3.14) to find the response frequencies of the modes.

For the case where *r*=3, i.e. *r*_1_=1 and *r*_2_=3, the resonant equations of motion may be written

3.16a $$4(\omega_{n1}^{2}-\omega^{2})U_{1}+3\alpha_{1}U_{1}^{3}+2\alpha_{3}U_{1}U_{2}^{2}+3p\alpha_{2}U_{1}^{2}U_{2}=0$$

and

3.16b $$4(\omega_{n2}^{2}-9\omega^{2})U_{2}+2\alpha_{3}U_{1}^{2}U_{2}+3\alpha_{5}U_{2}^{3}+p\alpha_{2}U_{1}^{3}=0.$$

Assuming *U*_1_≠0 and *U*_2_≠0 as before, equations (3.16) may be written as

3.17a $$\omega^{2}=\omega_{n1}^{2}+\frac{1}{4}[3\alpha_{1}U_{1}^{2}+2\alpha_{3}U_{2}^{2}+3\alpha_{2}U_{1}U_{2}e^{+j{\hat{\phi }}_{3,1}}]$$

and

3.17b $$\omega^{2}=\frac{\omega_{n2}^{2}}{9}+\frac{1}{36}[2\alpha_{3}U_{1}^{2}+3\alpha_{5}U_{2}^{2}+\alpha_{2}U_{1}^{3}U_{2}^{-1}e^{-j{\hat{\phi }}_{3,1}}],$$

and combined to give the amplitude relationship

3.18 $$\left[-p\frac{\alpha_{2}}{9}U_{2}^{-1}\right]U_{1}^{3}+\left[\frac{27\alpha_{1}-2\alpha_{3}}{9}\right]U_{1}^{2}+[3p\alpha_{2}U_{2}]U_{1}+\left[4\left(\omega_{n1}^{2}-\frac{\omega_{n2}^{2}}{9}\right)+\frac{6\alpha_{3}-\alpha_{5}}{3}U_{2}^{2}\right]=0,$$

which provides approximate solutions to the fundamental amplitudes for the case, where *r*=3.

If *r*≠1,3, such that *δ*_1_=*δ*_3_=0, the resonant equations of motion are written as

3.19a $$4(\omega_{n1}^{2}-r_{1}^{2}\omega^{2})U_{1}+3\alpha_{1}U_{1}^{3}+2\alpha_{3}U_{1}U_{2}^{2}=0$$

and

3.19b $$4(\omega_{n2}^{2}-r_{2}^{2}\omega^{2})U_{2}+2\alpha_{3}U_{1}^{2}U_{2}+3\alpha_{5}U_{2}^{3}=0.$$

These expressions are distinct from equations (3.13) and (3.16) as they do not contain phase-dependent terms. This implies that, for any response where *r*≠1,3 (such as the 3 : 8, 4 : 11 and 4 : 13 branches considered earlier) the phase-difference between the fundamental components of the modes may assume any value. Following the terminology introduced in [29], these are denoted the *phase-unlocked* cases. The cases where *r*=1 and *r*=3, however, require specific phase-differences between the modes, i.e. *ϕ*_1_−*ϕ*_2_=0,*π* and 3*ϕ*_1_−*ϕ*_2_=0,*π* respectively—these are described as the *phase-locked* cases. Recalling that only the conditionally resonant terms lead to phase relationships in the resonant equations of motion, it follows that phase-locking will only occur when the specific conditions for resonance are met, such as *r*=1,3.

Following the same procedure as the phase-locked cases, equation (3.19) may be written in terms of the base frequency

3.20 $$\omega^{2}=\frac{\omega_{n1}^{2}}{r_{1}^{2}}+\frac{1}{4r_{1}^{2}}[3\alpha_{1}U_{1}^{2}+2\alpha_{3}U_{2}^{2}]=\frac{\omega_{n2}^{2}}{r_{2}^{2}}+\frac{1}{4r_{2}^{2}}[2\alpha_{3}U_{1}^{2}+3\alpha_{5}U_{2}^{2}],$$

and amplitude relationship

3.21 $$4\left(\frac{\omega_{n1}^{2}}{r_{1}^{2}}-\frac{\omega_{n2}^{2}}{r_{2}^{2}}\right)+\left[\frac{3\alpha_{1}}{r_{1}^{2}}-\frac{2\alpha_{3}}{r_{2}^{2}}\right]U_{1}^{2}+\left[\frac{2\alpha_{3}}{r_{1}^{2}}-\frac{3\alpha_{5}}{r_{2}^{2}}\right]U_{2}^{2}=0,$$

which may be solved to find the phase-unlocked responses in terms of the amplitudes and response frequencies of the underlying linear modes.

**Figure 6.**
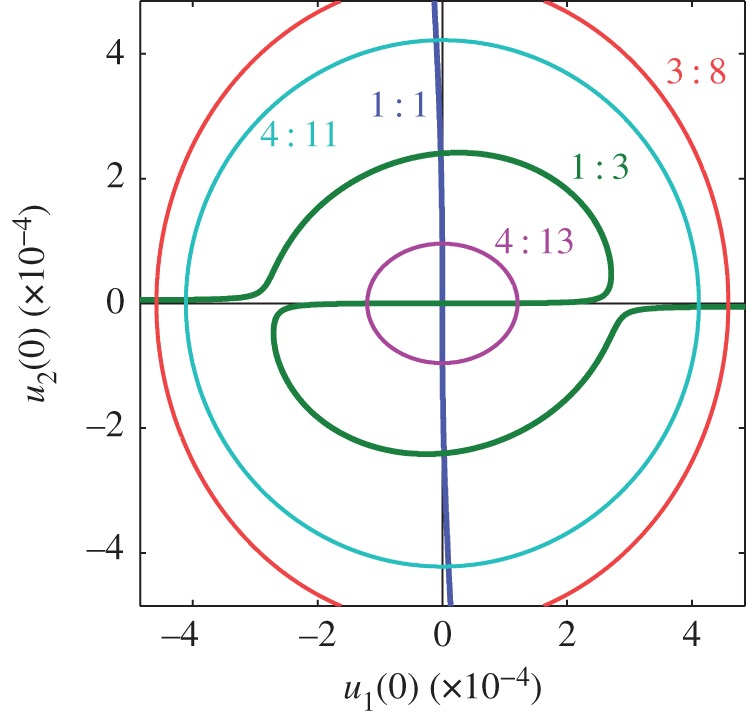
Five NNM branches, computed using the second-order normal form technique. These are represented in terms of the fundamental components of the linear modes at *t*=0. Thick lines are used to represent the phase-locked branches (1 : 1 and 1 : 3), while phase-unlocked branches (3 : 8, 4 : 11 and 4 : 13) are represented by thin lines. The colour scheme used in previous figures is adopted. (Online version in colour.)

Figure 6 shows the NNM branches of the two-mode beam model, computed using the second-order normal form technique. This clearly resembles figure 3*a*, where the branches are computed using numerical continuation. In figure 6, the phase-locked responses (1 : 1 and 1 : 3) are represented by thick lines and the phase-unlocked responses (3 : 8, 4 : 11 and 4 : 13) are represented by thin lines. Note that the 1 : 1 branch only shows responses that are in anti-phase, i.e. sgn(*u*_1_(0))=−sgn(*u*_2_(0)), found when *p*=−1. Although the 1 : 1 case does yield solutions when *p*=+1, using equations (3.14) and (3.15), these solutions lead to harmonic components that are greater than the fundamentals within the region shown here. As the fundamental components are defined as those with the greatest amplitude, these solutions are rejected. Note that figure 6 shows only the initial values of the fundamental component, and the harmonics are not included.

In [29], the NNM branches were computed for different models of a symmetric, taut horizontal cable. It was shown that both phase-locked^5^ and phase-unlocked branches exist for these models and, using an analytical stability analysis, it was demonstrated that internal resonance in the forced responses may follow the phase-locked branches, but not the phase-unlocked branches. Based on this observation, we now consider the difference between the behaviour of these two classes of NNM branch. Specifically, our hypothesis is that the phase-unlocked branches may be neglected when the NNMs are used to interpret the forced responses of a system. The stability analysis presented in [29] exploits the special properties exhibited by the system due to its symmetry and hence cannot readily be generalized to asymmetric systems, such as the beam considered here. As such, a different approach is required to investigate the significance of the phase-unlocked NNMs. In the following section, the phase difference between the modes is investigated and an energy-based approach is used to explore the relationship between the forced responses and the NNMs.

## 4. Phase considerations

In this section, the influence of the phase difference between the underlying linear modes of the system are explored. Firstly, in §4a, a numerical method is employed to investigate the relationship between the forced responses of the system and the NNMs. This is used to develop a measure of *attractiveness* of the forced responses and the NNMs. Next, in §4b, the analytical expressions derived in §3 are used to show that the phase difference between the modes permits energy to be transferred within the system—a feature that is essential for many forced responses. This allows the difference in the energy transfer mechanisms of phase-locked and phase-unlocked responses to be compared, and gives insight into why these classes of NNM exhibit different relationships with the forced responses.

### (a) Perturbing a forced response on a nonlinear normal mode

In many engineering applications, nonlinear normal modes are used to develop an understanding of the forced responses of systems. This is based on the assumption that the forced responses are *attracted* to the NNMs [25]. In order to investigate this attraction, we now consider the case where a damped version of the two-mode beam model is forced such that the response is precisely that of an NNM solution. A delay is then applied to the external forcing in one of the modes, and the resulting change in the steady-state response of the system is measured. It is assumed that NNMs require a specific phase difference between the linear modes; therefore, if a delay in the forcing leads to a change in the phase of the response, the response is no longer that of an NNM. Furthermore, if an NNM is highly attractive it is expected that the change in response, resulting from the phase perturbation, will be small. However, if the attraction is weak, the change in the response is expected to be of a similar order to the perturbation. These responses are computed numerically, employing a time-domain integration scheme and a shooting algorithm to find the steady-state perturbed solutions.

If external forcing and linear modal damping are applied to the beam, the equation of motion takes the form

4.1 $$\ddot{\text{q}}+\zeta \dot{\text{q}}+\Lambda \text{q}+{\text{N}}_{q}(\text{q})=\text{f},$$

where ***ζ*** is a linear modal damping matrix and **f** is a modal forcing vector. Comparing equations (2.3) and (4.1) reveals that if the forcing equals the damping, i.e. $\text{f}=\zeta \dot{\text{q}}$, then the response will precisely match that of an NNM. This is denoted as *normal forcing*. This section considers the case where a delay (or shift in phase), *δ*, is applied to one element of the normal forcing such that

4.2 $$\left(\;\begin{array}{c} f_{1}(t)\\ f_{2}(t) \end{array}\right)=\left(\begin{array}{c} 2\zeta \omega_{n1}{\dot{q}}_{1}(\tau T)\\ 2\zeta \omega_{n2}{\dot{q}}_{2}([\tau +\delta ]T) \end{array}\right),$$

where *τ*=*t*/*T* and *ζ* is the linear modal damping ratio (assumed to be equal for both modes). This delay will perturb the forced response from the NNM solution, and it is assumed that the perturbed response may be expressed in terms of the NNM solutions with a time delay (or phase), i.e. ${\bar{q}}_{i}(t)=q_{i}([\tau +\varepsilon_{i}]T)$, where ${\bar{q}}_{i}$ and *q*_*i*_ denote the perturbed and NNM solutions, respectively. As such, the change in the phase between the two modes may be expressed *ε*_*d*_=*ε*_2_−*ε*_1_. For a sufficiently small *δ*, the gradient of *ε*_*d*_ with respect to *δ* may be estimated as

4.3 $$\frac{d\varepsilon_{d}}{d\delta }\approx \frac{\varepsilon_{d}^{+}-\varepsilon_{d}^{-}}{2|\delta |},$$

where $\varepsilon_{d}^{+}$ and $\varepsilon_{d}^{-}$ are the values of *ε*_*d*_ when δ=+∣δ∣ and δ=−∣δ∣, respectively.

**Figure 7.**
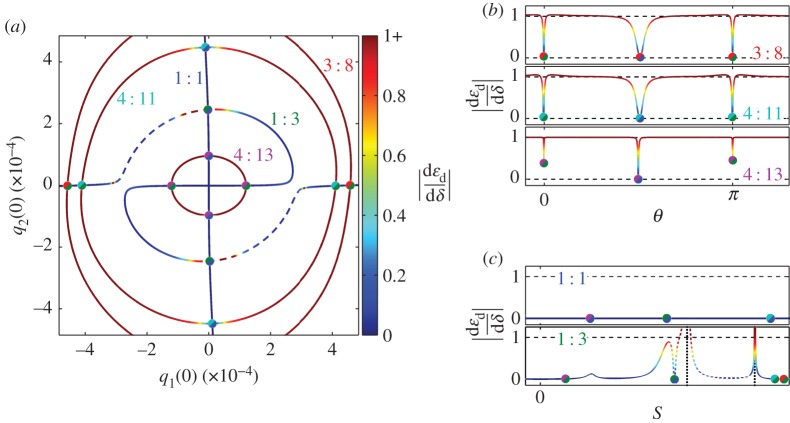
The phase gradients of the five NNM branches, previously considered in figure 3. Panel (*a*) shows the NNM branches in the projection of the initial displacements of the modes, *q*_1_(0) against *q*_2_(0). The absolute values of the phase gradients of these NNMs are represented by a colour gradient, as shown by the colour bar. As in previous figures, the branches are labelled, and the unstable section of the 1 : 3 branch is dashed. To aid comparison between the panels, the intersection points between the branches are marked using coloured circles. Panel (*b*) shows the 3 : 8, 4 : 11 and 4 : 13 branches in the projection of *θ* against the absolute phase gradient, $|d\varepsilon_{d}/d\delta |$, where *θ* denotes the angle in the *q*_1_(0) against *q*_2_(0) plane. The 1 : 1 and 1 : 3 branches are shown in panel (*c*) in the projection of *S* against $|d\varepsilon_{d}/d\delta |$, where *S* is the arc length in the *q*_1_(0) against *q*_2_(0) plane. The horizontal dashed lines in panels (*b*) and (*c*) mark the phase gradients at 0 and 1, and the vertical dotted lines in (*c*) show the asymptotes. (Online version in colour.)

Figure 7 shows the phase gradients on the NNM branches considered previously for the example beam. These have been computed for the case where the system has linear damping with *ζ*=0.01. Figure 7*a* shows the NNM branches in the projection of the initial displacements of the modes, *q*_1_(0) against *q*_2_(0), as used previously in figure 3*a*. A colour gradient is used to represent the absolute phase gradient, $|d\varepsilon_{d}/d\delta |$, as shown by the colour bar on the r.h.s. of the plot. Note that the upper limit of this colour bar is 1+, as some gradients exceed 1. In the projection of *q*_1_(0) against *q*_2_(0), the 3 : 8, 4 : 11 and 4 : 13 branches are near-circular. This property is exploited in figure 7*b*, where the phase gradients of the NNMs of these branches are shown in the projection of the angle (in the *q*_1_(0) against *q*_2_(0) plane), *θ*, against the absolute phase gradient, $|d\varepsilon_{d}/d\delta |$. Similarly, figure 7*c* shows the phase gradient of the 1 : 1 and 1 : 3 branches. As these are not circular, the projection of the arc length (in the *q*_1_(0) against *q*_2_(0) plane), *S*, against the absolute phase gradient, $|d\varepsilon_{d}/d\delta |$, is used. Owing to the symmetry of these NNM branches, only half of the branches are shown, i.e. between 0 and *π* for figure 7*b* and between 0 and 0.5 for figure 7*c* (where *S*=0 marks *q*_1_(0)=*q*_2_(0)=0 for these branches). Note that the colour scheme used in figure 7*a* is also employed in figures 7*b*,*c*. A series of coloured circles (as used previously in figure 3) are used in figure 7 to show the points where the NNM branches intersect in the *q*_1_(0) against *q*_2_(0) projection.

Figure 7 demonstrates how the phase gradients differ between the NNM branches. Firstly, it can be seen that the 1 : 1 branch has a gradient close to 0 at all points shown here. This shows that the responses exhibit little change when the external forcing is perturbed, suggesting that these NNMs are highly attractive to the forced responses. As such, it may be concluded that the 1 : 1 branch is a key feature for using NNMs to interpret the forced responses of this system.

The 1 : 3 branch shows a more complex behaviour and it can be seen that this behaviour is related to the unstable regions (represented by dashed lines). This includes the presence of vertical asymptotes, represented by vertical, dotted black lines in figure 7*c*. However, aside from the regions in the vicinity of the unstable NNMs, the phase gradient for this branch is close to zero. This suggests that the influence of the stable portion of the 1 : 3 branch is similar to that of the 1 : 1 branch, and represents a key feature underlying the forced responses.

It can be seen that the phase-unlocked (i.e. the 3 : 8, 4 : 11 and 4 : 13 branches) have phase gradients close to 1 at most points. This shows that the majority of NNMs on these branches do little to attract the forced responses, as the change in the response is of the same order as the perturbation. As such, the phase-unlocked branches are expected to have little influence on the forced responses. It can also be seen that, in vicinity of the crossing points with phase-locked branches, the phase gradient of the phase-unlocked branches decreases towards zero. This is to be expected as, at the crossing points, the two branches must share solutions, and hence must also share phase-gradients. Furthermore, as phase-unlocked branches approach a crossing point, they must converge to that of a phase-locked response. For example, at the crossing point with the 1 : 3 branch, the 3 : 8 branch must be exhibiting a 3 : 9 response. As such, it is expected that in vicinity of the crossing point (where the phase gradient is seen to decrease) the ninth harmonic of the second mode grows to be larger than the fundamental. This analysis suggests that the phase-locked branches are influential structures underpinning the forced responses of this system, whereas the majority of phase-unlocked solutions have less influence.

### (b) Energy transfer between modes

It has been shown in §4a that phase-locked and phase-unlocked NNM branches have differing degrees of influence on the forced responses. We now build upon the results presented in [29], showing internally resonant behaviour in a taut cable. In [29], it was demonstrated that phase-unlocked branches do not correspond to internally resonant responses (where a mode that is not directly forced exhibits a response). Internal resonance requires a net energy transfer from the forced mode into the unforced mode (assuming the unforced mode is losing energy to damping). This energy transfer thus represents a physical mechanism that must be present in order for a system to exhibit internal resonance. As such, in this section, this energy transfer mechanism is investigated for both phase-locked and phase-unlocked responses.

The net energy transfer into the *i*th linear mode over one period of motion may be written as

4.4 $$E_{i}=\int_{0}^{T}N_{qi}(t){\dot{q}}_{i}(t)\;\mathrm{dt},$$

where *N*_*qi*_ represents the nonlinear terms in the *i*th conservative equation of motion. Neglecting the harmonics, this may be approximated to

4.5 $$E_{i}=\int_{0}^{T}N_{ui}(t){\dot{u}}_{i}(t)\;\mathrm{dt},$$

where *N*_*ui*_ represents the nonlinear terms in the *i*th resonant equation of motion. As these nonlinear terms all respond at the *i*th fundamental frequency, *ω*_*ri*_, this may be written as

4.6a $$\begin{array}{rl} E_{i} & =-U_{i}\omega_{ri}\int_{0}^{T}\left\{\sum_{k=1}^{K_{i}}{\bar{N}}_{ui,k}\cos[\omega_{ri}t-(\phi_{i}+\psi_{i,k})]\right\}\sin(\omega_{ri}t-\phi_{i})\;\mathrm{dt}, \end{array}$$

4.6b $$\begin{array}{rl} & =-\pi U_{i}r_{i}\sum_{k=1}^{K_{i}}{\bar{N}}_{ui,k}\sin(\psi_{i,k}), \end{array}$$

where ${\bar{N}}_{ui,k}$ and *ψ*_*i*,*k*_ represent the amplitude and phase of the *k*th term in *N*_*ui*_, respectively; *K*_*i*_ denotes the total number of terms in *N*_*ui*_; and where the assumed solution for *u*_*i*_, equation (3.2), has been used. Recalling the form of the resonant equations of motion, equations (3.8) and (3.9), phase-locking may only occur when *ψ*_*i*,*k*_≠0 and, for phase-unlocked responses, *ψ*_*i*,*k*_=0 for all terms. As such, equation (4.6b) demonstrates that, for phase-unlocked responses, a shift in phase will not lead to a transfer of energy between the resonant components of the underlying linear modes.

Although net energy transfer does not occur between modes in a steady-state conservative response (such as an NNM) it may occur in forced and transient responses. In the case of internal resonance, as considered in [25], a net energy transfer from the forced mode to the unforced mode must be present if the unforced mode is to exhibit a response (assuming the unforced mode is damped). As such, if an internally resonant forced response is close to an NNM, that NNM must be able to transfer energy between the modes. This explains why no internally resonant responses were seen to follow the phase-unlocked backbone curves in [29], as no energy can be transferred via the fundamental components of the response.

This analysis also helps explain the difference between the phase gradients of the phase-locked and phase-unlocked branches shown in figure 7—the change in the external forcing will change the net energy transfer into each mode due to the forcing. To compensate for this, the net energy must either be transferred between the linear modes or the modes must alter their response to reduce this change. The phase-locked branches are able to exhibit a small shift in phase to accommodate the transfer, whereas the phase-unlocked branches cannot, and so must alter their response phase to match that of the forcing. However, the analysis presented here only considers the energy via the fundamental components. Next, we explore the role of harmonics in the transfer of energy between modes.

### (c) Harmonics of a nonlinear normal mode

The second-order normal form technique, used earlier to compute analytical expressions for the NNM branches, may also be used to compute analytical expressions describing the harmonics. This has previously been demonstrated in [30,34], for example. Recalling equations (3.6) and (3.7), the matrix of harmonic coefficients, [*h*], may be computed. Using this, along with **h**=[*h*]u*, allows the harmonics of the first and second modes to be written

4.7a $$h_{1}=\sum_{k=1}^{7}H_{1,k}\cos[(\mu_{k}+\nu_{k}r)(\omega_{r1}t-\phi_{1})+\nu_{k}{\hat{\phi }}_{r,1}]$$

and

4.7b $$h_{2}=\sum_{k=1}^{7}H_{2,k}\cos[(\mu_{k}+\nu_{k}r^{-1})(\omega_{r2}t-\phi_{2})+\nu_{k}r^{-1}{\hat{\phi }}_{r,1}],$$

where

$$\begin{array}{rl} \mu_{1} & =0,\;\mu_{2}=0,\;\mu_{3}=1,\;\mu_{4}=1,\;\mu_{5}=2,\;\mu_{6}=2,\;\mu_{7}=3,\\ \nu_{1} & =1,\;\nu_{2}=3,\;\nu_{3}=-2,\;\nu_{4}=2,\;\nu_{5}=-1,\;\nu_{6}=1,\;\nu_{7}=0, \end{array}$$

and where the amplitudes of these components, *H*_1,*k*_ and *H*_2,*k*_, are listed in appendix B.

The presence of the phase-difference, ${\hat{\phi }}_{r,1}=r\phi_{1}-\phi_{2}$, in these expressions demonstrates that, even when phase-locking is not present in the resonant equations of motion (i.e. when *r*=1,3), the harmonics are phase-dependent. As such, it may be assumed that the phase-unlocked responses may transfer energy via the harmonics when there is a change in the phase-difference between the modes.

As the harmonics are smaller than the fundamental components of the response they may only transfer a small amount of energy. Therefore, the phase-locked responses (which are able to transfer energy via the fundamental components) may transfer more energy than the phase-unlocked responses (which may only transfer via the harmonics). This demonstrates a fundamental difference between the underlying mechanisms of these two classes of NNM, and it may be concluded that it is the relative weakness of this energy-transfer mechanism that prevents many forced responses from following the phase-unlocked branches, as observed in [29]. In applications where the harmonics are assumed to be negligible, it is reasonable to assume that the phase-unlocked NNMs transfer a negligible amount of energy between the modes.

## 5. Forced responses

In previous sections, it has been argued that phase-unlocked NNM branches have little influence on the forced responses as the energy may only be transferred between the linear modes of the system via the action of the harmonics. In this section, the case where a sinusoidal forcing is applied to the second linear mode is considered. Initially, damping levels that are typical of engineering structures are used, and it is shown that the phase-locked NNM branches are followed. Next, an extremely low damping level is used, and it is shown that this allows the phase-unlocked branches to be followed. This demonstrates that phase-unlocked branches may be followed by the forced responses when the damping is very low, as the required energy transfer between the linear modes is sufficiently small.

**Figure 8.**
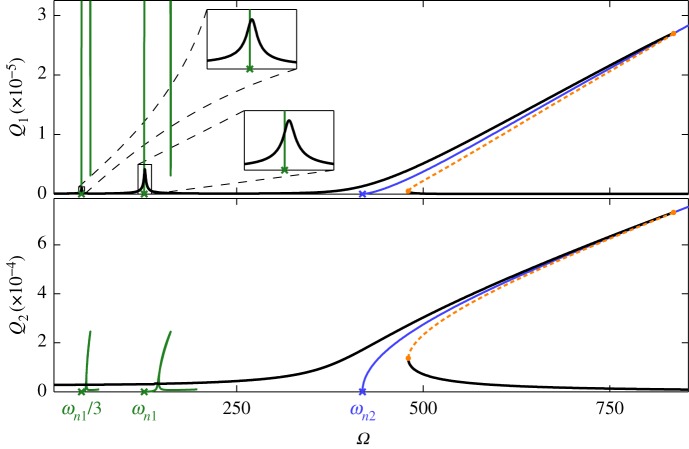
The NNM and forced response branches when the second mode is forced with an amplitude of *P*=5 and where the damping ratio is *ζ*=0.01. These forced responses are shown in the projection of the forcing frequency, *Ω*, against the maximum displacements of the first and second modes, *Q*_1_ and *Q*_2_, respectively. The stable and unstable forced responses are represented by solid-black and dashed-orange lines respectively, and fold bifurcations are represented by orange dots. The NNM branches are represented by solid-blue and solid-green lines, and crosses mark the linear natural frequencies, as labelled. Two regions, near to *Ω*=*ω*_*n*1_/3 and *Ω*=*ω*_*n*1_, are shown in detail in the *Ω* against *Q*_1_ projection. (Online version in colour.)

The sinusoidal forcing considered in this section is only applied to the second linear mode such that, recalling the forced equations of motion from equation (4.1), this is expressed as *f*_1_=0 and $f_{2}=P\cos(\Omega t)$ (where *P* is the forcing amplitude and *Ω* is the forcing frequency). As previously, a linear modal damping is assumed. Figure 8 shows the 1 : 1 and 1 : 3 NNM branches, along with an additional 1 : 3 branch at one third of the base frequency such that *Ω*=*ω*/3. A segment of this branch, referred to as the *subharmonic* 1 : 3 branch, has previously been shown in figure 3*c*, intersecting with the 3 : 8 NNM branch. Note that, as with the bottom panel in figure 3*c*, the complete loop in the 1 : 3 and subharmonic 1 : 3 branches cannot be seen. To help simplify this figure, the 1 : 3 and subharmonic 1 : 3 branches are truncated in the *Ω* against *Q*_2_ projection.

Along with the NNM branches, figure 8 also shows forced responses when the forcing amplitude is *P*=5 and the linear damping ratio is *ζ*=0.01. It can be seen that a large peak envelops the 1 : 1 NNM branch and a smaller peak envelops the 1 : 3 branch. This is to be expected as, at low amplitude, the 1 : 1 branch is primarily composed of the second mode (the forced mode) while the 1 : 3 branch is primarily composed of mode 1 (figure 3*a*). As shown in detail in figure 8, a small peak also envelops the subharmonic 1 : 3 branch.^6^ It can be expected that decreasing the damping ratio, or increasing the forcing amplitude, will lead to a growth in the forced response following this subharmonic branch, and the response following the 1 : 1 branch will become extremely large. Figure 8 also shows that the peaks do not envelop the NNM branches precisely—they appear to be shifted to a higher frequency. This is due to the relatively high amplitude reached by the second mode away from resonance, which leads to a stiffening effect in the system, as described in [29,37].

**Figure 9.**
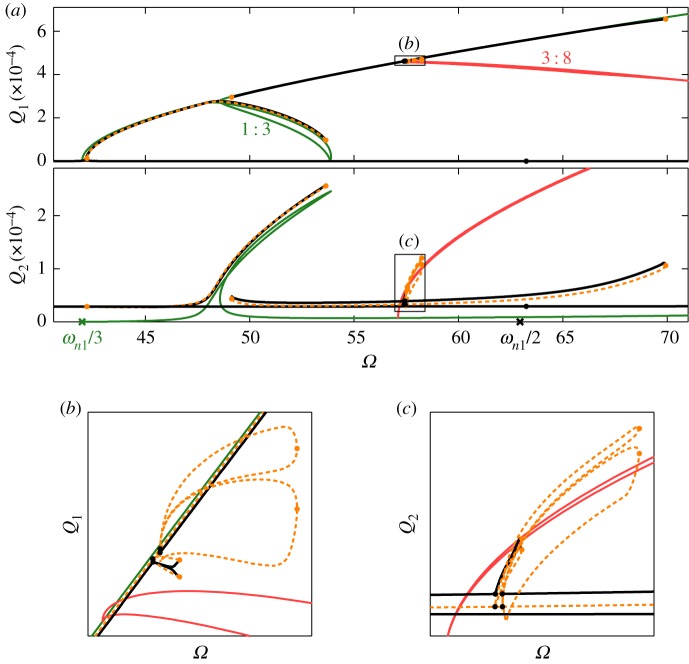
The NNM and forced response branches when the second mode is forced with an amplitude of *P*=5 and where the damping ratio is *ζ*=10^−8^. Panel (*a*) shows the forced responses in the projection of the forcing frequency, *Ω*, against the maximum displacements of the first and second modes, *Q*_1_ and *Q*_2_, respectively. Note that the range of forcing frequencies is smaller than that shown in figure 8. The NNM branches are represented by solid lines, using the colour scheme adopted for previous figures. The stable and unstable forced responses are represented by solid-black and dashed-orange lines, respectively, while the fold and branch point bifurcations are represented by orange dots and black dots, respectively. The boxes, labelled (*b*) and (*c*), mark the regions that are shown in detail in panels (*b*) and (*c*). (Online version in colour.)

Figure 9 shows the forced responses when the system is forced with an amplitude of *P*=5 and with a damping ratio of *ζ*=10^−8^—an equal forcing but significantly lower damping ratio than that used in figure 8. While figure 8 shows the forced responses over a forcing frequency range including the first and second linear natural frequencies, *ω*_*n*1_ and *ω*_*n*2_, the range in figure 9 is much smaller, and details the responses enveloping the 1 : 3 subharmonic branch. The very low damping considered here leads to an extremely high-amplitude response enveloping the 1 : 1 branch, and hence is not shown here. Figure 9*a* shows, in the *Ω* against *Q*_2_ projection, that the second mode exhibits a non-zero response away from resonance, due to the external forcing. As previously shown in figure 8, this causes a slight shift in the frequency of the forced responses.

Figure 9*a* shows that, near *ω*_*n*1_/3, the first linear mode exhibits a response that follows the 1 : 3 subharmonic NNM branch very closely. This response follows the loop region of the branch before reaching a fold bifurcation. A similar behaviour is observed in the second mode, although an amplitude shift is also introduced by the forcing. Along with this primary forced response branch, an isola^7^ is observed, enveloping the upper section of the 1 : 3 subharmonic NNM branch. Close to where the subharmonic 1 : 3 and 3 : 8 NNM branches meet, a set of branch point bifurcations connect the isola to a set of additional responses. These responses are shown in detail in figure 9*b*,*c*, where they appear to follow the subharmonic 3 : 8 branch. An additional branch point bifurcation can be seen near *Ω*=*ω*_*n*1_/2, which is found to lead to an additional set of responses following the subharmonic 1 : 3 branch at *Ω*=*ω*/2. As this subharmonic branch does not connect to any phase-unlocked branches, this response is not shown.

**Figure 10.**
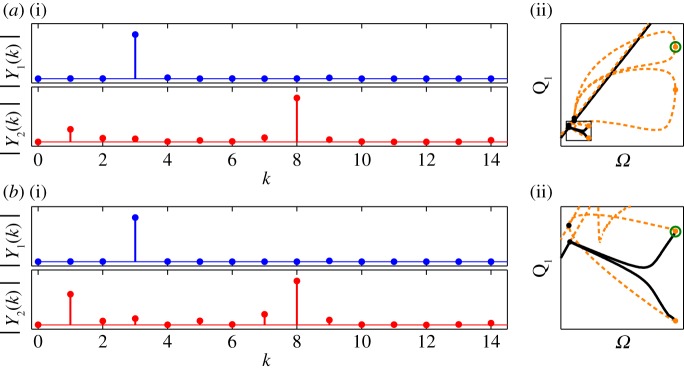
The amplitudes of the first 15 Fourier components of two different solutions on the forced response branches following the 3 : 8 NNM branch. Panel (*a*(ii)) shows the larger of the two forced response branches in detail, in the projection of forcing frequency against the amplitude of displacement of the first mode, and a green circle marks the response whose Fourier components are shown in panel (*a*(i)). A black rectangle in (*a*(ii)) shows the region that is depicted in panel (*b*(ii)), showing the smaller of the two forced response branches. The green circle in this panel marks the response whose Fourier components are shown in panel (*b*(i)). (Online version in colour.)

Figure 10 shows the amplitudes of the first 15 Fourier components at two points on the forced responses following the subharmonic 3 : 8 NNM branch. As shown in figure 10*a*(ii), figure 10*a*(i) shows the Fourier components at a fold bifurcation on the larger of the two sets of responses, while figure 10*b*(i) shows the components at a fold bifurcation on the smaller of the two. In both cases, the fundamental component of the first mode (represented by the blue dots in the upper plots) is at three times the forcing frequency, while the fundamental component of the second mode (red dots in the lower plots) is at 8*Ω*. There is also a fairly large component in the second mode responding at the forcing frequency, due to the external forcing acting directly on the second mode. However, this clearly shows that in both cases the responses are exhibiting a 3 : 8 response.

This demonstrates that the forced responses may follow the 3 : 8 NNM branch, determined to be phase-unlocked in the analytical analysis. Fundamentally, the system is only forced in the second mode, and so a 3 : 8 response (in which the first mode is active and damped) requires a net transfer of energy from the second to the first mode. This shows that the 3 : 8 response is able to transfer energy between the modes. Furthermore, the external forcing is at a single harmonic and well away from the linear natural frequencies. As such, in exhibiting a 3 : 8 response, the system is resisting the external forcing and this response is driven by its attraction to the 3 : 8 NNMs.

The 3 : 8 forced responses may only be observed when the damping is extremely low (*ζ*=10^−8^ in the case considered here). As such, only a small amount of energy is lost from the first, unforced, mode, and hence only a small amount of energy must be transferred between the modes. This net energy transfer is sufficiently small that it may be achieved via the harmonics, which is required for a phase-unlocked NNM branch to be followed. If the damping is increased to *ζ*=2.7×10^−6^, it is found that the isola disappears, and hence the mechanism for reaching the 3 : 8 branch no longer exists. This is still an extremely low level of damping, and rarely seen in mechanical structures.

Therefore, while it has been demonstrated in this section that the phase-unlocked NNMs are able to attract forced responses, they do not appear to do so in the majority of engineering systems, with more conventional levels of damping. If the damping is extremely low, the forced responses may be attracted to the phase-unlocked NNMs, resulting in a large number of complex resonant behaviours. However, these resonances are likely to be much less significant than those enveloping the phase-locked branches.

## 6. Conclusion

This paper has considered the dynamic behaviour of a simple nonlinear model of a beam. It has been shown that this system may exhibit a large number of NNM branches, i.e. the loci of responses of the underlying conservative system. These branches describe a vast array of dynamic behaviours, many of which are not observed when the system is forced and damped. As NNMs are often used to gain an understanding of the forced responses, this highlights the need for a method for determining which NNM branches relate to the forced responses, and which can be neglected.

To gain an understanding of the mechanisms that govern the NNMs, the dynamics of the NNM branches was described using an analytical method. This revealed two distinct classes of NNM branch: phase-locked branches, where the fundamental components of the responses were coupled via specific phase relationships; and phase-unlocked branches, where no phase relationship between the fundamental components is directly enforced. This phase relationship is key to understanding how the NNMs correspond to the forced responses.

The influence of the phase relationship between the modes was investigated by considering a perturbation to the phase of the external forcing. The forcing was initially such that the response was precisely that of an NNM, and hence the perturbation leads to a deviation of the response from the NNM. The magnitude of this deviation was used as a measure of the attraction between the NNM and the forced response. This assumes that a strongly attractive NNM will be robust to the external forcing, and hence the deviation will be small, while a forced response on a weakly attractive NNM will deviate further. The results of this analysis demonstrated that the phase-locked NNM branches are strongly attractive, while the phase-unlocked NNMs are weaker.

To explain the nature of attraction between the NNMs and forced responses, the energy transfer between the linear modes of the system was then considered. This revealed that the phase-locked NNMs are able to transfer energy via the fundamental components of the modes, while the phase-unlocked NNMs can only transfer energy via the harmonics. As the harmonics are typically much smaller than the fundamental components, this mechanism for energy transfer is much weaker in phase-unlocked responses.

The importance of the energy transfer mechanism is then demonstrated by considering the case where the system is sinusoidally forced. When the damping is very low, the net energy transfer between the modes is sufficiently small that it may be achieved by some phase-unlocked NNMs. However, when the damping is at a level that is more typical of engineering structures, the required energy transfer is too great and the phase-unlocked NNM branches cannot be followed. This was demonstrated using two different damping values: one that is typical of engineering structures, where the phase-unlocked branches are not followed; and one extremely low value, where a phase-unlocked branch is followed. It is therefore concluded that phase-unlocked NNMs will only influence the forced responses in systems with extremely low damping, and may be disregarded in other cases. This reduces the number of NNM branches that need to be considered, and simplifies their use as a tool for understanding forced responses.

## Supplementary Material

Conditionally-resonant terms and phase dependence

## Footnotes

1 Throughout this paper, the term *mode* refers to an underlying linear mode of the system, unless otherwise specified. A *nonlinear normal mode* (NNM) is a separate concept, and is typically composed of multiple linear modes.

2 Note that *ω*_*ri*_ and *ω*_*ni*_ are distinct and represent the fundamental response frequency (i.e. the frequency of the component with the highest amplitude) and linear natural frequency of the *i*th mode, respectively.

3 Note that the conditions $r=\frac{1}{3}$, *r*=1 or *r*=3 are specific to this system and, if alternative nonlinearities were considered, conditionally resonant terms may arise from other values of *r*. For example, it is expected that quadratic nonlinearities may lead to 1 : 2, or *r*=2, resonances.

4 A copy of this proof is provided as the electronic supplementary material to this paper.

5 Note that the phase-locked branches represent a whirling in the cable which, as discussed in [12], is an out-of-unison motion.

6 For further discussion of subharmonic resonances, see [36].

7 An isola is a detached section of the forced response. For further discussion of isolas, see [26,28].

## Data accessibility

The data presented in this work are openly available from the University of Bristol repository at http://dx.doi.org/10.5523/bris.18ov1ijiiox0u1b0z40rzcsnwl.

## Appendix A. Parameters of the beam model

From [32], the modeshape of the *i*th linear mode may be written as

A 1 $$\theta_{i}(x)=\sqrt{2}{\left[1-2\frac{EI}{\hat{k}L}\sin^{2}(\beta_{i})-{\left(\frac{\sin(\beta_{i})}{\sinh(\beta_{i})}\right)}^{2}\right]}^{-1/2}\left[\sin\left(\frac{\beta_{i}}{L}x\right)-\frac{\sin(\beta_{i})}{\sinh(\beta_{i})}\sinh\left(\frac{\beta_{i}}{L}x\right)\right],$$

where *β*_*i*_ may be found by solving

A 2 $$\cot(\beta_{i})-\coth(\beta_{i})+2EI\frac{\beta_{i}}{\hat{k}L}=0.$$

Additionally, the linear natural frequencies may be found using

A 3 $$\omega_{ni}^{2}=\frac{EI}{\rho \hat{A}}{\left(\frac{\beta_{i}}{L}\right)}^{4},$$

and the nonlinear coefficients are found from

A 4 $$\begin{array}{l} \alpha_{1}=\frac{-E}{2L^{2}\rho }\;\left[\int_{0}^{L}\theta_{1}^{\prime }\theta_{1}^{\prime }\;\text{dx}\right]\left[\int_{0}^{L}\theta_{1}^{\prime \prime }\theta_{1}\;\text{dx}\right],\;\alpha_{\text{2}}\text{ = }\frac{-E}{\text{2}{\text{L}}^{\text{2}}\rho }\left[\int_{\text{0}}^{L}\theta_{\text{1}}^{\prime }\theta_{\text{1}}^{\prime }\;\text{dx}\right]\left[\int_{\text{0}}^{L}\theta_{\text{1}}^{\prime \prime }\theta_{\text{2}}\;\text{dx}\right],\\ \alpha_{\text{3}}\text{ = }\frac{-E}{\text{2}L^{\text{2}}\rho }\left\{\text{2}\left[\int_{\text{0}}^{L}\theta_{\text{1}}^{\prime }\theta_{\text{2}}^{\prime }\;\text{dx}\right]\left[\int_{\text{0}}^{L}\theta_{\text{2}}^{\prime \prime }\theta_{\text{1}}\;\text{dx}\right]\text{ + }\left[\int_{\text{0}}^{L}\theta_{\text{2}}^{\prime }\theta_{\text{2}}^{\prime }\;\text{dx}\right]\left[\int_{\text{0}}^{L}\theta_{\text{1}}^{\prime \prime }\theta_{\text{1}}\;\text{dx}\right]\right\},\\ \alpha_{\text{4}}\text{ = }\frac{-E}{\text{2}L^{\text{2}}\rho }\left[\int_{\text{0}}^{L}\theta_{\text{2}}^{\prime }\theta_{\text{2}}^{\prime }\;\text{dx}\right]\left[\int_{\text{0}}^{L}\theta_{\text{2}}^{\prime \prime }\theta_{\text{1}}\;\text{dx}\right]\;\text{and}\;\alpha_{\text{5}}\text{ = }\frac{-E}{\text{2}L^{\text{2}}\rho }\left[\int_{\text{0}}^{L}\theta_{\text{2}}^{\prime }\theta_{\text{2}}^{\prime }\;\text{dx}\right]\left[\int_{\text{0}}^{L}\theta_{\text{2}}^{\prime \prime }\theta_{\text{2}}\;\text{dx}\right]. \end{array}\}$$

## Appendix B. Amplitudes of the harmonic components

The amplitudes of the harmonics components, described in equations (4.7), may be computed using

B 1 $$\begin{array}{rl} H_{1,1} & =\frac{3\Delta_{1}}{r+1}\left(\frac{\alpha_{2}}{2}U_{1}^{2}U_{2}+\frac{\alpha_{4}}{4}U_{2}^{3}\right),\;H_{1,2}=\frac{\alpha_{4}U_{2}^{3}}{4(3r+1)(3r-1)},\\ H_{1,3} & =\frac{\alpha_{3}\Delta_{1}}{16r}U_{1}U_{2}^{2},\;H_{1,4}=\frac{\alpha_{3}U_{1}U_{2}^{2}}{16r(r+1)},\;H_{1,5}=\frac{3\alpha_{2}}{4}\Delta_{1}\Delta_{3}U_{1}^{2}U_{2},\\ H_{1,6} & =\frac{3\alpha_{2}U_{1}^{2}U_{2}}{4(r+1)(r+3)},\;H_{1,7}=\frac{\alpha_{1}}{32}U_{1}^{3}, \end{array}\}$$

and

B 2 $$\begin{array}{rl} H_{2,1} & =\frac{-3\Delta_{1}}{r+1}\left(\frac{\alpha_{2}}{4}U_{1}^{3}+\frac{\alpha_{4}}{2}U_{1}U_{2}^{2}\right),\;H_{2,2}=\frac{-\alpha_{2}\Delta_{3}U_{1}^{3}}{4(r+3)},\;H_{2,3}=\frac{-\alpha_{3}}{16}\Delta_{1}U_{1}^{2}U_{2},\\ H_{2,4} & =\frac{\alpha_{3}U_{1}^{2}U_{2}}{16(r+1)},\;H_{2,5}=\frac{3\alpha_{4}\Delta_{1}U_{1}U_{2}^{2}}{4(3r-1)},\;H_{2,6}=\frac{3\alpha_{4}U_{1}U_{2}^{2}}{4(r+1)(3r+1)},\\ H_{2,7} & =\frac{\alpha_{5}U_{2}^{3}}{32r^{2}} \end{array}\},$$

where *Δ*_*i*_ is defined by

B 3 $$\Delta_{i}=\left\{ \begin{array}{ll} 0 & \text{if:}\;i=r,\\ {\left(r-i\right)}^{-1} & \text{if:}\;i\neq r. \end{array}\right.$$

Note that it has been assumed that $r=\frac{1}{3}$ is not a solution.
