# Supplementary material for: Identifying the significance of nonlinear normal modes
Source: Proc Math Phys Eng Sci. 2017 Mar;473(2199):20160789. doi: 10.1098/rspa.2016.0789 (PMC5378240; doi:10.1098/rspa.2016.0789)
Supplement: Conditionally-resonant terms and phase dependence [file rspa20160789supp1.pdf]

Supplementary material for *Identifying the significance of nonlinear normal modes*

T.L. Hill, A. Cammarano, S.A. Neild and D.A.W. Barton

Conditionally-resonant terms and phase dependence

For an N-DOF system, the ℓth element of the vector of variables, $\mathbf{u}^*$, may be written

$$u_\ell^* = \prod_{k=1}^{N} u_{pk}^{s_{p\ell,k}} u_{mk}^{s_{m\ell,k}} , \tag{0.1}$$

where u_{pk} and u_{mk} are defined as

$$u_{pk} = \frac{U_k}{2} \exp\left\{+\mathrm{j}\left(\omega_{rk}t - \phi_k\right)\right\}, \qquad u_{mk} = \frac{U_k}{2} \exp\left\{-\mathrm{j}\left(\omega_{rk}t - \phi_k\right)\right\}, \tag{0.2}$$

where U_k, ω_{rk} and ϕ_k represent the amplitude, response frequency and phase of the fundamental component of the kth linear mode respectively.

Substituting Eqs. (0.2) into Eq. (0.1) gives

$$u_\ell^* = \left[\prod_{k=1}^{N} \left(\frac{U_k}{2}\right)^{\left(s_{p\ell,k} + s_{m\ell,k}\right)}\right] \exp\left\{+\mathrm{j} \sum_{k=1}^{N} \tilde{s}_{\ell,k} \left(\omega_{rk}t - \phi_k\right)\right\}, \tag{0.3}$$

where $\tilde{s}_{\ell,k} = s_{p\ell,k} - s_{m\ell,k}$. Using this, element $\{i,\, \ell\}$ of $\boldsymbol{\beta}$ may be found using

$$\beta_{i,\ell} = \left(\sum_{k=1}^{N} \tilde{s}_{\ell,k}\omega_{rk}\right)^2 - \omega_{ri}^2 , \tag{0.4}$$

where $\beta_{i,\ell}$ represents a resonant term when $\beta_{i,\ell} = 0$. We now define r_k such that $\omega_{rk} = r_k\omega$, where ω represents the base frequency i.e. $\omega = 2\pi/T$ and where T is the period of the response. Substituting this into Eq. (0.4), $\beta_{i,\ell}$ represents a resonant term when

$$\left(\sum_{k=1}^{N} \tilde{s}_{\ell,k} r_k\right)^2 = r_i^2 . \tag{0.5}$$

If the term represented by this is unconditionally-resonant, then Eq. (0.5) must be satisfied regardless of the values of r_k. It can be seen that this is only true when

$$\tilde{s}_{\ell,k} = \begin{cases} 0 & \text{for } k \neq i , \\ \pm 1 & \text{for } k = i . \end{cases} \tag{0.6}$$

For a conservative system, the vector of resonant nonlinear terms, $\mathbf{N}_u$, is written

$$\mathbf{N}_u\left(\mathbf{u}\right) = \left[N_u\right] \mathbf{u}^*, \tag{0.7}$$

where $[N_u]$ is an $\{N \times L\}$ matrix of coefficients whose $\{i,\, \ell\}^{\mathrm{th}}$ element is written $[N_u]_{i,\ell}$. Therefore the ith element of $\mathbf{N}_u$ is given by

$$N_{ui} = \sum_{\ell=1}^{L} \left[N_u\right]_{i,\ell} u_\ell^* . \tag{0.8}$$

Using Eq. (0.3), this may be written

$$N_{ui} = \sum_{\ell=1}^{L} K_{i,\ell} \exp\left\{+\mathrm{j} \sum_{k=1}^{N} \tilde{s}_{\ell,k} \left(\omega_{rk}t - \phi_k\right)\right\} . \tag{0.9}$$

If all terms are unconditionally-resonant (i.e. there are no conditionally-resonant terms in the resonant equation of motion) then, using Eq. (0.6), Eq. (0.9) may be written

$$N_{ui} = \sum_{\ell=1}^{L} [N_u]_{i,\ell} \left[\prod_{k=1}^{N} \left(\frac{U_k}{2} \right)^{\left(s_{p\ell,k} + s_{m\ell,k}\right)} \right] \mathrm{e}^{\pm\mathrm{j}\,(\omega_{ri} t - \phi_i)}, \tag{0.10}$$

The ith resonant equation of motion is written

$$\ddot{u}_i + \omega_{ni}^2 u_i + N_{ui} = 0\,, \tag{0.11}$$

which, substituting the assumed solution for u_i and Eq. (0.10), gives

$$\left\{ \left(\omega_{ni}^2 - \omega_{ri}^2\right) \frac{U_i}{2} + \sum_{\ell=1}^{L} [N_u]_{i,\ell} \left[\prod_{k=1}^{N} \left(\frac{U_k}{2} \right)^{\left(s_{p\ell,k} + s_{m\ell,k}\right)} \right] \right\} \mathrm{e}^{\pm\mathrm{j}\,(\omega_{ri} t - \phi_i)} = 0\,. \tag{0.12}$$

The time-independent components of Eq. (0.12) may then be written

$$\left(\omega_{ni}^2 - \omega_{ri}^2\right) \frac{U_i}{2} + \sum_{\ell=1}^{L} [N_u]_{i,\ell} \left[\prod_{k=1}^{N} \left(\frac{U_k}{2} \right)^{\left(s_{p\ell,k} + s_{m\ell,k}\right)} \right] = 0\,. \tag{0.13}$$

It is clear that Eq. (0.13) is independent of phase. This demonstrates that, if a resonant equation of motion contains only unconditionally-resonant terms (i.e. it does not contain any conditionally-resonant terms), the solution will be phase-unlocked. As such, it may be concluded that only conditionally-resonant terms may lead phase-locking terms, and hence conditionally-resonant terms are required for phase-locked NNMs.
